# PPNEMA: A Resource of Plant-Parasitic Nematodes Multialigned Ribosomal Cistrons

**DOI:** 10.1155/2008/387812

**Published:** 2008-09-23

**Authors:** Francesco Rubino, Amalia Voukelatou, Francesca De Luca, Carla De Giorgi, Marcella Attimonelli

**Affiliations:** ^1^Dipartimento di Biochimica e Biologia Molecolare, Università di Bari, Via E. Orabona 4, 70126 Bari, Italy; ^2^Sezione di Bari, Istituto per la Protezione delle Piante del CNR, Via Amendola 165, 70126 Bari, Italy

## Abstract

Plant-parasitic nematodes are important pests of crop plants worldwide, and also among the most difficult animals to identify. Their identification based on nuclear ribosomal DNA (rDNA) cistron (18S, 28S, and 5.8S RNA genes, and internal transcribed spacers, ITS1 and ITS2) is becoming a popular tool. Sequences from nuclear ribosomal RNA repeats have been used to demonstrate the identity of isolates from various hosts and to unravel the relationships of cryptic and complex species. In addition, the availability of RNA sequences allows study of phylogenetic relationships between nematodes, also for more complete understanding of their biology as agricultural pests. PPNEMA is a *plant-parasitic nematode* bioinformatic resource. It consists of a database of ribosomal cistron sequences from various species grouped according to nematode genera, and a search system allowing data to be extracted according to both text and pattern searching. PPNEMA offers to the scientific community a preprocessed archive of plant parasitic nematode sequences useful for nematologists. It is a tool to retrieve plant nematode multialigned sequences for phylogenetic studies or to recognize a nematode by comparing its rDNA sequence with the PPNEMA available genus specific multialignments.

## 1. INTRODUCTION

Plant-parasitic nematodes are devastating parasites of crop plants, reducing the overall yield or lowering the market value of crops [1, 2]. Nematodes are remarkably consistent in their anatomy [3], and their identification is essentially based on morphometric characters. In addition, as variations occur in host responses to attack by various morphologically indistinguishable populations of several parasitic species, correct species identification is fundamental for efficient nematode control. For this reason, direct examination of genetic material has, recently, been used as it represents the most powerful method for nematodes recognition.

Although phytoparasitic nematodes have evolved specific structures for their survival as parasites, these adaptations are essentially built around a basic framework of nematode anatomy. Many biological questions can thus be addressed by placing the nematode *Caenorhabditis elegans,* the best characterised multicellular organism [4], in a phylogenetic and evolutionary context, together with plant-parasitic nematodes.

**Figure 1:**
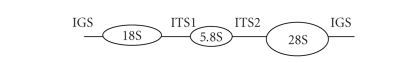
*rDNA cistron scheme*. Representation of nematode ribosomal RNA genes arranged in tandemly repeated clusters: rDNA array.

The nucleotide sequences of fragments of rRNA genes have recently been obtained in various species of plant-parasitic nematodes, yielding a proper platform for both identification and taxonomic approaches [5]. Nematode ribosomal RNA genes are arranged in tandemly repeated clusters (rDNA arrays) containing the genes for 18S, 5.8S, and 26S ribosomal RNA, separated by internal transcribed spacers ITS1 and ITS2 and bordered by IGS intergenic spacers (see Figure 1). Only few sequences available in the primary nucleotide databases span the entire rDNA array, although in several cases phylogenetic relationships within different species of plant-parasitic nematodes have been obtained even when only fragments of ribosomal genes were used [6–8].

This paper describes the PPNEMA database, grouping and analysing rRNA genes sequenced in plant-parasitic nematodes and present in the primary databases. It should be noted that, although specific and important nematode resources are available on the web, such as WormBase ([9], http://www.wormbase.org/), Nematode.net ([10], http://www.nematode.net/), NemATOL (http://nematol.unh.edu/index.php), the Comprehensive Phytopathogen Genome Resource (CPRG) (http://cpgr.tigr.org/index.html), NEMrRNA ([11], http://www.nemamex.ucr.edu/rna/), and NEMBASE ([12], http://www.nematodes.org/nematodeESTs/nembase.html) a database resource for nematode EST datasets. The last three contain sequences from rRNA genes and therefore are likely to be of interest to any reader of this article. However, the innovative aspect of PPNEMA is the availability of the rDNA sequences in groups of multialigned sequences.

## 2. MATERIALS AND METHODS

### 2.1. Data source

Sequence data are derived from primary databases (EMBL/GenBank/DDBJ) using the retrieval systems SRS and Entrez. Since a single entry in the primary database can contain more elements of the same cistron, the extraction of the sequences of each element is supported by the information contained in the entry's features table. Moreover, in order to reduce false negatives obtained through the retrieval system, the extracted data are compared to the whole database by applying the Blast database similarity searching system. In this way, sequences of interest for PPNEMA (plant parasitic nematode rDNA sequences) are found, which are not correctly annotated in primary databases and which hence are lost during the text searching retrieval.

### 2.2. Software

Extracted sequences are analysed by applying (i) the CleanUP software [13] which allows the detection of redundant sequences, and (ii) the ClustalW [14] software which produces the genus/cistron_element specific multialignment. Data so obtained are stored in the PPNEMA database. The database is physically based on MySQL DBMS [15], and the web application is based on an application framework written in Python.

## 3. RESULTS

### 3.1. Aim of PPNEMA

The aim of PPNEMA is to offer end-users a ready to use compilation of multialigned plant-parasitic nematode ribosomal cistrons, of which thousands of sequences are available in primary nucleotide databases (EMBL/GenBank/DDBJ). The sequences of several rRNA regions retrieved from primary databases are analysed and stored in the PPNEMA database, grouped by each nematode genus. Thus, PPNEMA is a preprocessed archive of data ready to be used from researchers interested in phylogenetic studies on phytoparasitic nematodes, or to recognize a nematode by comparing its rDNA cistrons with the PPNEMA available genus specific multialigned groups.

### 3.2. Structure of PPNEMA database

**Figure 2:**
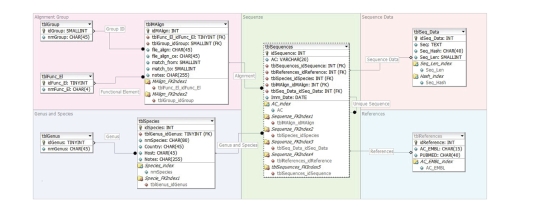
*PPNEMA relational database design*. It includes 8 tables, storing complete set of PPNEMA data linked to each other.

**Figure 3:**
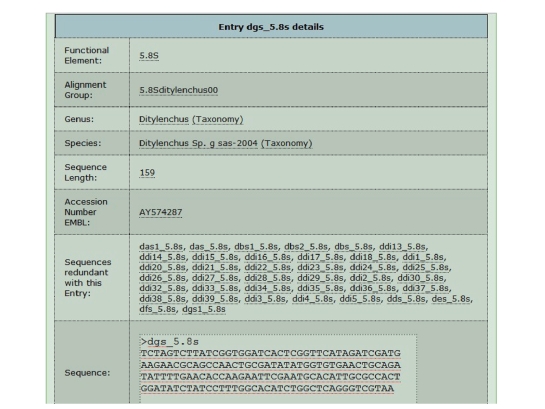
*Example of a PPNEMA entry*. Entry dgs_5.8S (PPNEMA ID) shows functional element, PPNEMA group ID to which entry belongs, Genus and Species names, sequence length, EMBL accession number, list of redundant sequences, and sequence, in FASTA format, which can be downloaded.

PPNEMA is a well-integrated, web-based, **plant-parasitic nematode** bioinformatics resource, allowing the storage, query, and analysis of phytoparasitic rDNA sequences. PPNEMA consists of a *database* of ribosomal cistron sequences from various species of plant-parasitic nematodes, grouped according to nematode genera and of a search system allowing data to be extracted according to both text and pattern searching. Each entry in the PPNEMA database refers to a complete or partial cistron element of a single isolate within a nematode species; it is identified by a code defining species and function. Sequences derived from the various species are multialigned within each nematode genus. However, since not all sequences span the entire rDNA array, separate multialignments have been produced for single rRNA genes or for portions of the same gene separately, depending on sequence availability. Each multialignment defines a group. The presence within a genus group of perfectly matching sequences (here defined as redundant) is determined by CleanUP software. Redundant sequences are stored in the database, linked to the group containing the group-reference sequence, but they are not enclosed in the multialignment of that group. Thus, each entry in the database is related to a species-specific functional element. Several entries are associated in a group. Several groups are available for the same genus and the same functional element. Figure 2 shows the database structure, and Figure 3 shows an example of a PPNEMA database entry.

### 3.3. Updating of PPNEMA database

Generally speaking, data in primary databases are organised in such a way that each entry is related to a genomic fragment of DNA related to a genome or one or more genes, complete or partial, so that the extraction of sequences related to the same cistronic element has been so far performed through, very time consuming, a nonautomated procedure. However, we have planned, but not yet implemented, a new updating procedure which will allow the automatic extraction from primary databases of the newly sequenced phytoparasites nematodes rDNA. The automatic procedure will generate one sequence for each entire or partial cistron element of a specific species; this sequence will be analysed through the application of the PPNEMA “characterizing” tool that will guide the automatic procedure in defining its better fitting multialignment group.

### 3.4. Contents of PPNEMA database

**Figure 4:**
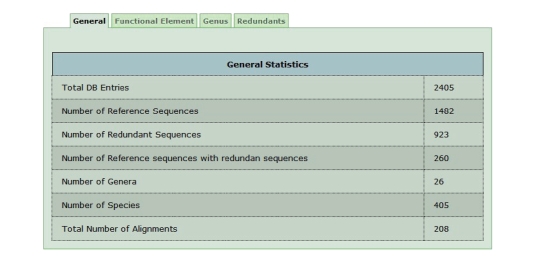
*General statistics* about PPNEMA data. It is also possible to obtain statistical information centred on functional elements or on redundant data content.

PPNEMA currently contains 2405 sequences, organised in 208 Alignment Groups from 26 genera. Because the plant-parasitic nematode RNA cistrons are not all conserved between and within genera, it is practically impossible to produce one multialignment for each element not only among all species but also among species of the same genera. This means that there are associated multialigned sequences in different groups for the same genera and, in order to have a reference, each multialignment was produced both with and without *Caenhorabditis elegans*, used as outgroup guide. More detailed information about database contents may be obtained through the Statistics option available through the PPNEMA site. Figure 4 shows data obtainable from the statistic option in PPNEMA.

### 3.5. Functions of PPNEMA

**Figure 5:**
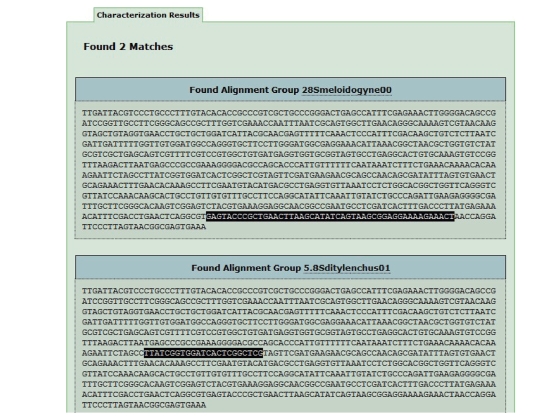
*Result of an anonymous sequence characterization*. The submitted sequence contains 2 fragments matching part of 28smeloidogyne00 and 5.8sditylenchus01 multialignment consensus.

Starting from the PPNEMA home page, two main options are available: search PPNEMA and browse PPNEMA. Both are organised in subsections. Search PPNEMA is used to retrieve specific sequences and/or aligned groups of sequences, through basic search, advanced search, or pattern search. Basic search allows retrieval of data according to the following criteria: functional element, genus name, species name, sequence length range. Advanced search allows more elaborate queries combining the various retrieval criteria through the logical operators AND or OR; selection criteria include the possibility to select data through a pattern searching option implemented on the basis of regular expressions. A regular expression is a powerful way of specifying a pattern for a complex search. The primer for the regular expressions used by MySQL is available through the help PPNEMA function. From the advanced search, a pattern search option is implemented within the search menu. The difference between the options “pattern search” and “pattern search through advanced search” is the output format of the retrieved data. Search results may be grouped by alignment, reference sequences, or redundancy groups. Retrieved sequences grouped by alignment are ready to be analysed with phylogenetic tools. Lastly, the option “characterising a new sequence” can search group/s of multialigned sequences, the consensus sequence of which, defined through regular expressions, matches submitted end-user sequence whose function and/or species paternity is undefined or not completely defined. Figure 5 shows an example of the output obtained by submitting a new sequence for its characterisation. The *browse DB* option allows the list of database species, multialignments, and sequences to be viewed. Starting from any element in the list, related information available in both PPNEMA and cross-referenced databases (e.g., EMBL, GenBank, and Taxonomy) can be obtained. Lastly, the PPNEMA resource contains online help, statistics tables, and an option, designed but not yet implemented, allowing submission of the new sequences on behalf of registered end-users. Registration is already implemented. In progress is the production of the phylogenetic trees, there where data which are variable enough to be informative by the evolutionary point of view. The produced trees will be available in the PPNEMA database.

## 4. CONCLUSIONS

The PPNEMA database is very helpful in identifying plant parasitic nematodes on a molecular basis, since the availability of multialigned sequences for nematode genera represents a map, on which the sequence of any unidentified nematode species can be located. In addition, the existence of several entries for the same species gives information on the extent of intraspecies variability and can thus help in discriminating between variants or new nematode species. This information is important in view of the expected rapid growth of sequence data from intraspecific studies aimed at both population migration and identification of different pathotypes.

It is important to emphasise that the more sequences obtained, the more informative the PPNEMA database will become. Periodical updating is foreseen, but contribution from sequence producers is welcome.

In conclusion, the perspective is extensive use of the PPNEMA database by plant pathologists who are not specialised in molecular biology.
